# ​Temporal proteomic profiling of iPSC-derived human liver organoids reveals optimal maturation for drug metabolism and toxicology

**DOI:** 10.1038/s41598-025-32539-0

**Published:** 2026-01-14

**Authors:** Shu Yang, Jon Hao, Masato Ooka, Menghang Xia

**Affiliations:** 1 Division for Pre-Clinical Innovation, National Center for Advancing Translational Sciences, National Institutes of Health, Rockville, MD USA; 2 Poochon Scientific, Frederick, MD USA

**Keywords:** iPSC-derived liver organoids, Proteomic analysis, Organoid development, CYP3A4, Protein expression, Biological techniques, Drug discovery, Medical research

## Abstract

Liver organoids have emerged as an innovative three-dimensional model system that effectively recapitulates the structural and functional complexities of human liver tissue, addressing limitations of traditional two-dimensional culture systems. This study presents a comprehensive temporal proteomic characterization of iPSC-derived human liver organoids (iHLOs) by profiling 5,736 proteins across their four developmental stages, Days 7, 15, 30, and 45, to identify optimal timepoints for drug metabolism and toxicological applications. The analysis revealed that Day 30 represents the peak of functional maturity, marked by the highest expression of key-metabolizing enzymes, such as cytochrome P450 3A4 (CYP3A4), along with cholangiocyte-specific proteins and hepatic metabolic enzymes. Functional validation of CYP3A4 activity through rifampicin induction and ketoconazole inhibition demonstrated metabolic competence comparable to existing in vitro liver models, including HepaRG cells and primary human hepatocytes. Interestingly, Day 45 organoids exhibited a shift toward a mesenchymal cell profile, with increased markers of hepatic stellate cells, suggesting utility for disease modeling applications. These findings indicate Day 30 of liver organoids as the optimal stage for drug metabolism and toxicological investigations, providing a proteomic framework that enhances their utility of iHLOs as physiologically relevant liver models.

**Supplementary Information:**

The online version contains supplementary material available at 10.1038/s41598-025-32539-0.

## Introduction

The liver is a vital organ that governs over hundreds of physiological processes, including nutrient metabolism^1^, detoxification^2^, bile secretion^3^, and protein synthesis^1^, serving as the body’s primary metabolic hub^4,5^. By neutralizing bloodborne chemicals and facilitating their excretion through bile or urine, the liver plays a critical role in maintaining systemic homeostasis^6^. Traditionally, in vitro liver studies have mainly relied upon two-dimensional (2D) cell culture models, by using primary hepatocytes or immortalized cell lines such as HepG2 and HepaRG cells. While these model systems provide valuable insights into liver function and drug metabolism, they fail to mimic the intricate three-dimensional (3D) architecture, cellular heterogeneity, and zonation observed in the native liver. Consequently, these models often insufficiently simulate the liver microenvironment, leading to discrepancies in drug metabolism and toxicity profiles and reducing their predictive accuracy for toxicological and pharmacological applications^7,8^. This inadequacy underscores the growing demand for more physiologically relevant in vitro liver models.

Organoids, defined as self-organized 3D structures derived from pluripotent stem cells or progenitors, have emerged as transformative tools for modeling human tissues such as the liver in vitro models^9,10^. Induced pluripotent stem cell (iPSC)-derived human liver organoids (iHLOs) are particularly promising, as they can recapitulate the cellular complexity of the liver, including diverse cell types such as hepatocytes, cholangiocytes, endothelial cells, and occasionally Kupffer cells^11^. These organoids offer invaluable applications, including disease modeling, drug metabolism studies, toxicity testing, and regenerative medicine. Furthermore, iHLOs enable patient-specific modeling, providing a platform for precision medicine tailored to individual genetic and pathological conditions^12,13^. Despite their utility, a significant challenge in iHLO research lies in determining the optimal developmental timepoints for liver-like functionality, which is critical for their effective use in pharmacological and toxicological studies. Variability in differentiation protocols and a lack of standardized benchmarks make it difficult to identify the most functionally matured and relevant stages of organoid development.

Proteomics, the large-scale study of protein expression, has emerged as a powerful tool for characterizing liver organoid maturation and functionality at the cellular and molecular level. By profiling protein expression dynamics, proteomics can offer a temporal roadmap of cellular differentiation and functional development in organoids^14–17^. Studies have confirmed that liver organoids express key enzymes, including members of the cytochrome P450 (CYP450) family and hepatic transporters, though often at lower levels compared to native liver tissue^14,15^. However, existing data on temporal coordination of protein expressions in iHLOs remain limited, and maturation timelines across different protocols appear inconsistent. These limitations highlight the need for systematic temporal studies to define the optimal stages of hepatic differentiation and functionality in iHLOs.

This study aims to address these gaps by conducting a comprehensive temporal proteomic analysis of iHLOs at four critical stages of development: Days 7, 15, 30, and 45. The differentiation protocol utilized was developed and optimized from the published literatures^18–21^, specifically chosen for its capacity to generate iHLOs with high CYP enzyme activity, particularly CYP3A4, which is crucial for drug metabolism and toxicology evaluations. These timepoints were selected to represent distinct stages of iHLO development and maturation: Day 7 signifies the hepatoblast differentiation stage, Day 15 marks the early hepatocyte stage, and Day 30 and Day 45 indicate progressively matured liver organoid stages with increasing liver cell populations and functions. The findings identify a robust pathway to hepatic maturation, with Day 30 emerging as the developmental peak characterized by optimal drug-metabolizing enzyme expression and functional hepatic features. Notably, this stage exhibits peak levels of CYP3A4, a major enzyme responsible for metabolizing 30–50% of clinically administered drugs^22,23^. Functional assays validate metabolic activity at Day 30, demonstrating comparable performance to widely used models such as primary human hepatocytes (PHHs) and HepaRG cells. In addition, later-stage organoids (Day 45) exhibit notable mesenchymal differentiation, expanding the potential applications of iHLOs to studies of complex liver pathologies such as fibrosis. By establishing a temporal framework for liver organoid development, this work facilitates the standardized application of iHLOs across drug screening, toxicology testing, and disease modeling.

## Results

### Temporal dynamics of protein profiles in iHLO development

**Fig. 1:**
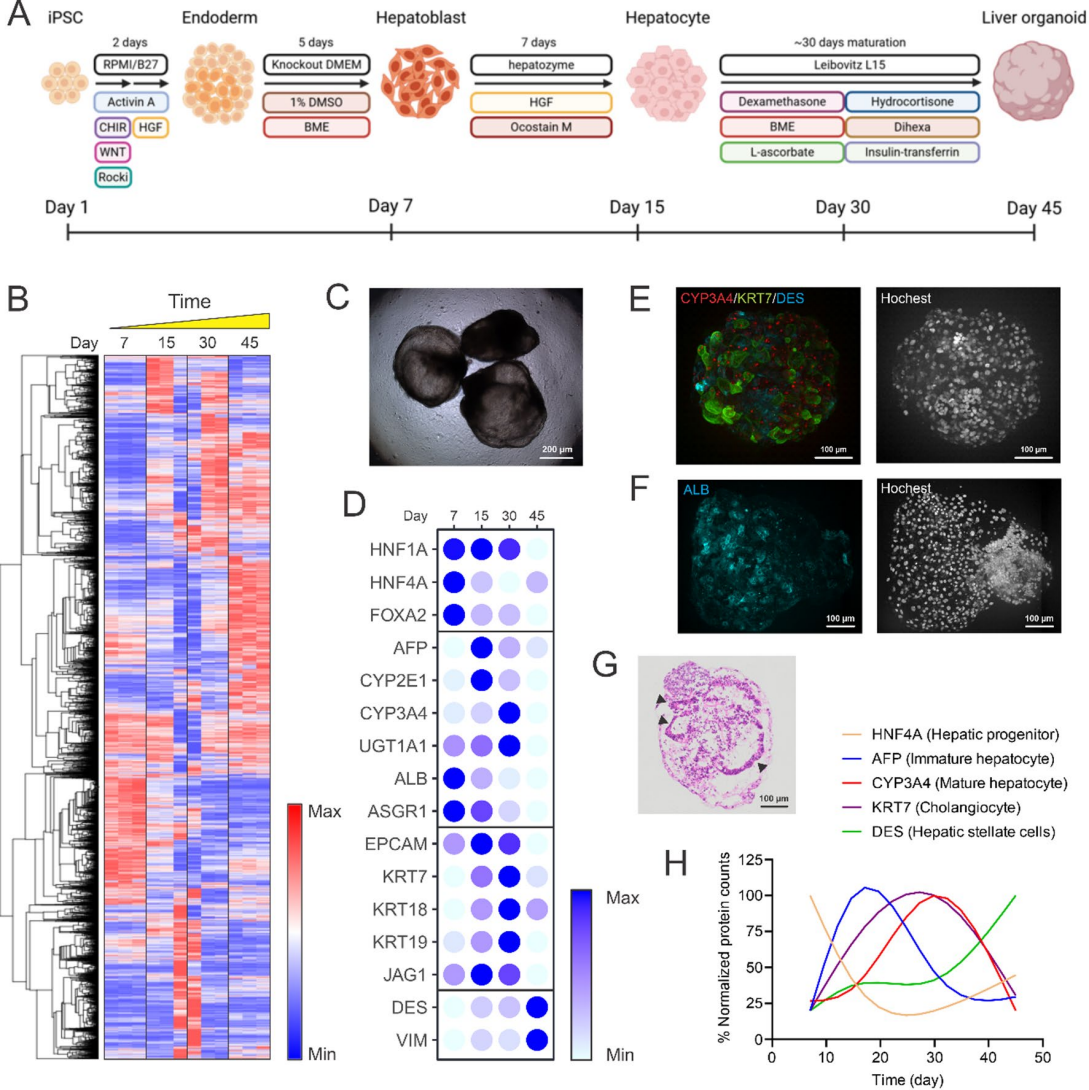
Temporal dynamics of protein profiles in iHLO development. (**A**) Schematic timeline illustrating the differentiation progression of iHLOs from iPSCs. (**B**) Heatmap visualization of proteomics data showing expression patterns of 5,736 proteins across four developmental timepoints (Days 7, 15, 30, and 45), with three biological replicates per timepoint. (**C**) The representative brightfield image of Day 30 iHLOs. (**D**) Expression of cell type-specific protein markers across the four developmental stages, highlighting temporal shifts in cellular composition. Bubble color intensity represents relative protein abundance. (**E**) Whole mount immunofluorescence staining of Day 30 iHLOs showing CYP3A4 (red), KRT7 (green), and DES (cyan) demonstrating co-differentiation of multiple cell lineages. (**F**) Whole mount immunofluorescence staining of Day 45 iHLOs showing ALB (cyan) localization predominantly on organoid surfaces. (**G**) H&E staining of Day 30 iHLOs revealing epithelial structures with distinct luminal formations. Arrowheads indicated luminal structure in the iHLOs. (**H**) Temporal expression profiles of five protein markers representing distinct liver cell populations throughout iHLO development, demonstrating the progressive maturation from progenitor to functionally diverse stages.

To elucidate the maturation trajectory of iHLOs, we analyzed the proteomic expression of 5,736 proteins across four developmental stages: Day 7, Day 15, Day 30, and Day 45 (Fig. 1A,B). Early-stage organoids (Day 7) exhibited a proteomic profile characteristic of hepatoblast-like populations, marked by high levels of stem cell-associated transcription factors such as hepatocyte nuclear factor 1 alpha (HNF1A), hepatocyte nuclear factor 4 alpha (HNF4A), and forkhead box protein A2 (FOXA2) (Fig. 1D). This finding is consistent with other reports that these genes’ expression typically peaks during early progenitor stage and decreases towards the final hepatocyte-like cells stage^24–26^. By Day 15, alpha-fetoprotein (AFP), a hallmark of immature hepatocytes, demonstrated a notable increase, coupled with the emergence of early hepatic metabolic proteins like CYP2E1, signaling the onset of hepatic differentiation (Fig. 1D).

As depicted in Fig. 1C, most Day 30 iHLOs consisted of epithelial cells forming a cystic structure. Proteomic analysis of Day 30’s iHLOs showed a decline in AFP expression, accompanied by a substantial upregulation of markers associated with mature hepatocytes such as CYP3A4 and UDP-glucuronosyltransferase 1A1 (UGT1A1). Moreover, cholangiocyte-associated proteins, including keratins 7 (KRT7) and 19 (KRT19), were prominently expressed. Immunostaining of Day 30 organoids confirmed the presence of CYP3A4 and albumin (ALB) in hepatocytes and KRT7 in cholangiocytes, indicating co-differentiation of these lineages (Fig. 1E and Supplementary 2 A). Additionally, desmin (DES) expression in a subset of cells identified hepatic stellate precursors in the population (Fig. 1E). Hematoxylin and eosin (H&E) staining revealed epithelial luminal structures, further substantiating epithelial maturation (Fig. 1G and Supplementary Fig. 1A)^27^. All these data suggest that iHLOs at Day 30 is in the peak of functional maturation.

By Day 45 of iHLOs, proteomic data signaled a transition toward mesenchymal differentiation. Significant upregulation of markers such as DES and vimentin (VIM) indicates an expansion of mesenchymal cell populations, including hepatic stellate-like cells or fibroblasts (Fig. 1D). This is further demonstrated by immunostaining of Day 45 iHLOs, which shows a significant increase and clustering of DES-expressing cells forming a solid structure distinct from the cystic structure composed of KRT7- or CYP3A4-expressing epithelial cells (Supplementary Fig. 2B). This indicates a zonation trend within iHLOSs with prolonged culturing. Concurrently, a decrease in mature hepatocyte-specific proteins like ALB was observed in proteomic assays (Fig. 1D), indicating increased cellular heterogeneity. However, immunostaining confirmed ALB cellular localization on organoid surfaces (Fig. 1F), suggesting the continued presence of mature hepatocytes and the formation of a cystic compartment in Day 45 iHLOs. This progression delineates a developmental pathway in iHLOs, transitioning from epithelial-centric populations to a more diverse composition including mesenchymal cell types by Day 45 (Fig. 1H).

### Differential protein expression patterns and pathway enrichment

**Fig. 2:**
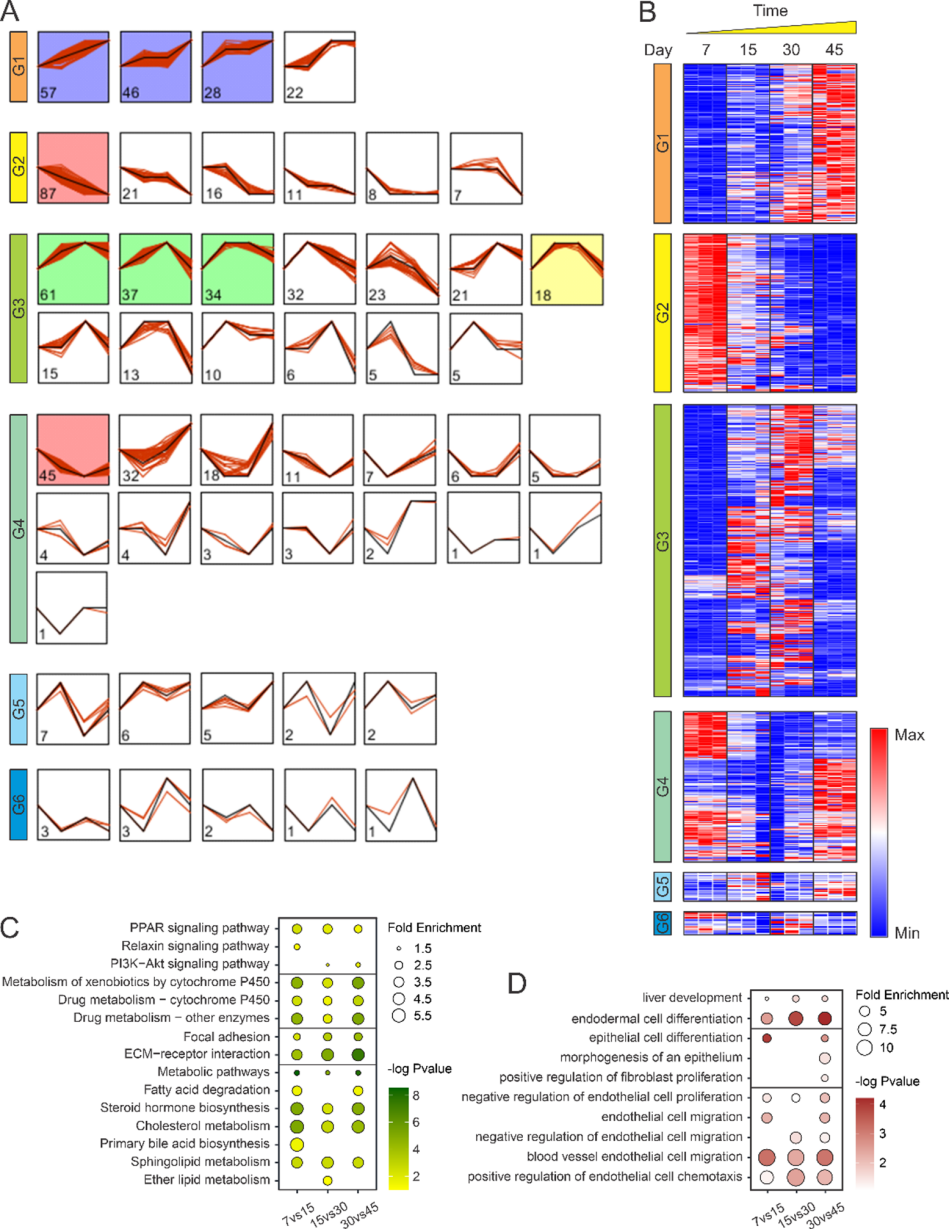
Differential protein expression patterns and pathway enrichment. (**A**) Classification of proteins into six distinct groups based on temporal expression patterns across four developmental stages using Short Time-series Expression Miner (STEM). The colored boxes indicate that the group contains a statistically significant number of assigned proteins. (**B**) Heatmap visualization of protein expression dynamics for each of the six pattern groups identified in panel A across all developmental timepoints. (**C**) KEGG pathway enrichment analysis of differentially expressed proteins during early (Days 7–15), mid (Days 15–30), and late (Days 30–45) developmental phases, highlighting temporal shifts in metabolic and signaling pathways. (**D**) GO biological process enrichment analysis of differentially expressed proteins during the three developmental phases, revealing stage-specific cellular processes. Dot size indicates the number of proteins in each pathway, while color intensity represents statistical significance (− log10 p-value).

Proteins were stratified into six distinct expression categories based on their temporal patterns across four timepoints: Group 1 (consistent increase), Group 2 (consistent decrease), Group 3 (increase then decrease), Group 4 (decrease then increase), Group 5 (up-down-up), and Group 6 (down-up-down) (Fig. 2A and B). Across these categories, three key developmental phases emerged: early (Days 7–15), mid (Days 15–30), and late (Days 30–45). This Temporal pattern-based analysis showed 660 differentially expressed proteins during the early phase (391 upregulated, 269 downregulated in Day 15), 543 during the mid phase (273 upregulated, 270 downregulated in Day 30), and 688 during the late phase (292 upregulated, 396 downregulated in Day 45) (Supplementary Table 1).

Pathway enrichment analysis *via* KEGG, using differentially expressed proteins in each phase, identified significant metabolic pathway enrichment throughout development (Fig. 2C and Supplementary Table 1). Lipid metabolism pathways, particularly steroid hormone biosynthesis and cholesterol metabolism, were highly enriched across all three iHLO development phases, indicating the organoids’ capacity to process lipids, especially cholesterol. Notably, primary bile acid biosynthesis was exclusively enriched at the early phase, suggesting a rapid development of bile acid production function in iHLOs at this stage. In addition, drug-metabolizing pathways, especially cytochrome P450, varied significantly across iHLO development, with highly enriched PPAR signaling, a pathway critical for hepatocyte differentiation and lipid metabolism. Concurrently, reduced statistical significance with the increased differentiation time indicates that the drug metabolism function becomes more stable as iHLOs mature. Focal adhesion and ECM-receptor interaction pathways exhibited sustained upregulation across all phases, highlighting enhanced extracellular matrix remodeling and cell-matrix communication as organoids matured. Furthermore, we observed enrichment of the relaxin signaling pathway at early iHLO phase, indicating an environment conducive to early liver epithelial cell differentiation. Activation of this pathway exhibits significant anti-fibrotic properties by inhibiting transforming growth factor-beta signaling and reducing the activation of hepatic stellate cells, which are key contributors to fibrosis. Moreover, PI3K-Akt signaling was significantly enriched at mid- and late phases of iHLO development, implying the expansion and functional maturation of the liver organoid, along with structural organization and functional integration of different cell types within the organoid.

In parallel, Gene Ontology (GO) analysis emphasized consistent enrichment in processes of endodermal cell differentiation and liver development across all three phases (Fig. 2D and Supplementary Table 1). Significant epithelial cell differentiation at the early phase supported the differentiation of hepatoblasts into hepatocytes at this stage. The significant epithelial cell differentiation at the late phase, coupled with the morphogenesis of an epithelium, indicated that epithelial morphogenesis of the liver occurred at this stage. This, along with increased fibroblast proliferation paralleling mesenchymal expansion, implies a major period of liver morphogenesis. Persistent endothelial cell proliferation and migration across all stages suggest uninterrupted endothelial lineage development, further contributing to organoid vascularization (Fig. 2D and Supplementary Table 1).

### Drug-metabolizing enzyme and transporter profiles

Given the importance of liver organoid models for studying xenobiotic metabolism, we profiled the enzymes and transporters essential for these functions. Proteomic analysis focused on 19 CYP450 enzymes, 36 non-CYP Phase I enzymes, 36 Phase II enzymes, and 95 drug transporters (16 ATP-binding cassette [ABC] transporters and 79 solute carrier [SLC] transporters).

**Fig. 3:**
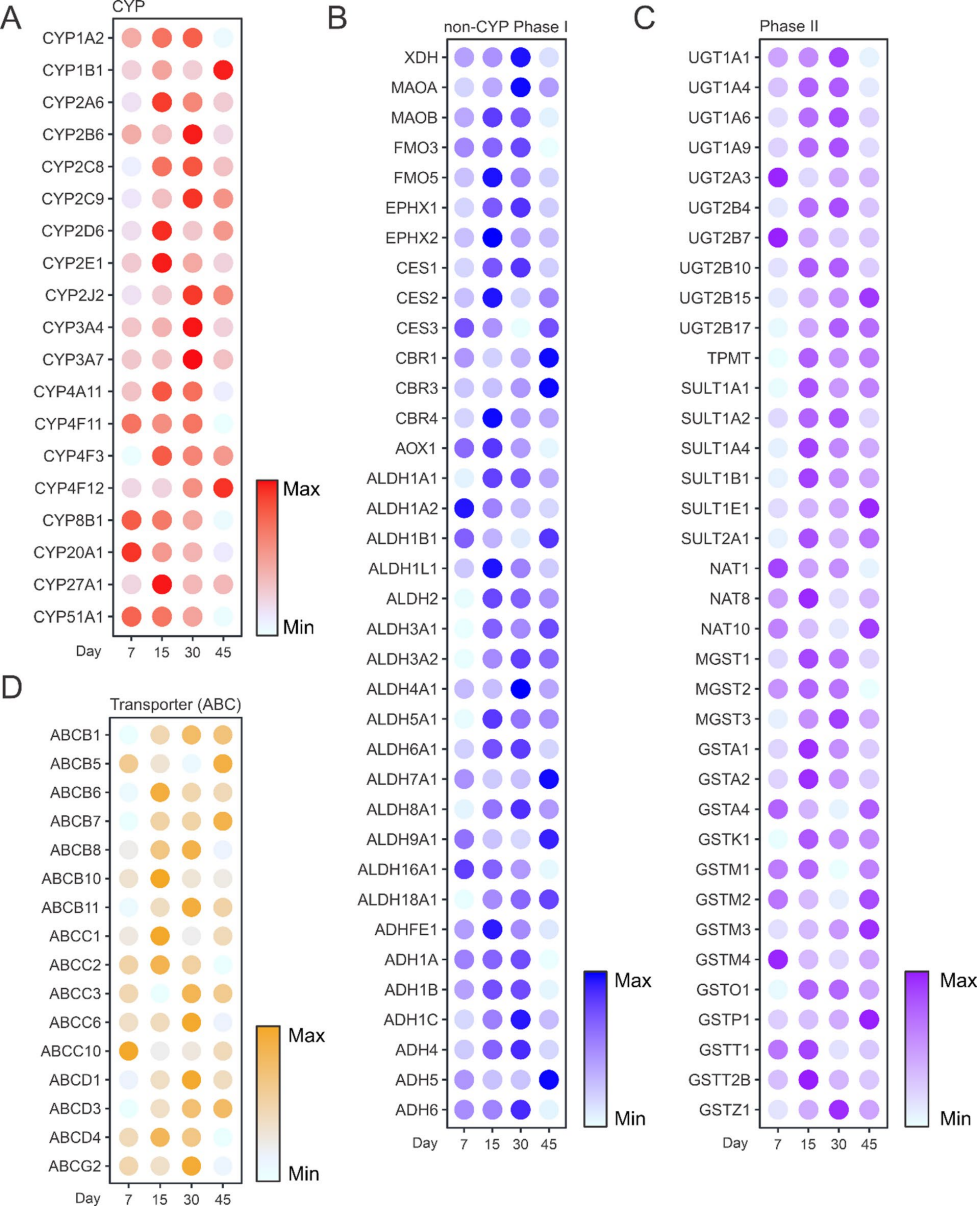
Drug-metabolizing enzyme and transporter profiles. Bubble plots depicting the expression levels of (**A**) CYP family enzymes, (**B**) non-CYP Phase I drug-metabolizing enzymes, (**C**) Phase II drug-metabolizing enzymes, and (**D**) ABC family transporters across four developmental stages (Days 7, 15, 30, and 45). Bubble color intensity indicates relative protein abundance.

Drug-metabolizing CYP450 enzymes, crucial for the liver’s role in processing drugs and other substances, exhibited peak expression between Day 15 and Day 30. Specifically, CYP3A4, CYP3A7, CYP2C9, and CYP2B6 reached maximal levels at Day 30 (Fig. 3A), with Western blot confirming higher CYP3A4 expression at Day 30 compared to earlier stages (Supplementary Fig. 2). By Day 45, reduced CYP expression correlated with the emergence of mesenchymal-like cells, although functional hepatocytes remained, as evidenced by continued ALB localization in immunostaining analyses. CYP1A2 displayed stable expression across Days 7–30, while CYP1B1 and CYP4F12 exhibited progressive increases, reaching maximum levels at Day 45, reflecting roles in fatty acid and bile acid metabolism during later stages.

Non-CYP Phase I and Phase II enzyme profiles mirrored these trends, peaking between Days 15 and 30 (Fig. 3B and C). Notably, enzymes such as aldehyde dehydrogenases (ALDHs) and glutathione S-transferases (GSTs) maintained relatively high expression at Day 45, possibly supporting metabolic adaptation to a diverse cellular environment. ABC transporters peaked at Day 30, with ABCB1 maintaining high expression on Day 45, emphasizing its role in drug efflux (Fig. 3D). Variable expression patterns were observed among SLC transporters, with some peaking as early as Day 15 and others persisting through Day 45 (Supplementary Fig. 3).

### iHLOs model for toxicology applications

**Fig. 4:**
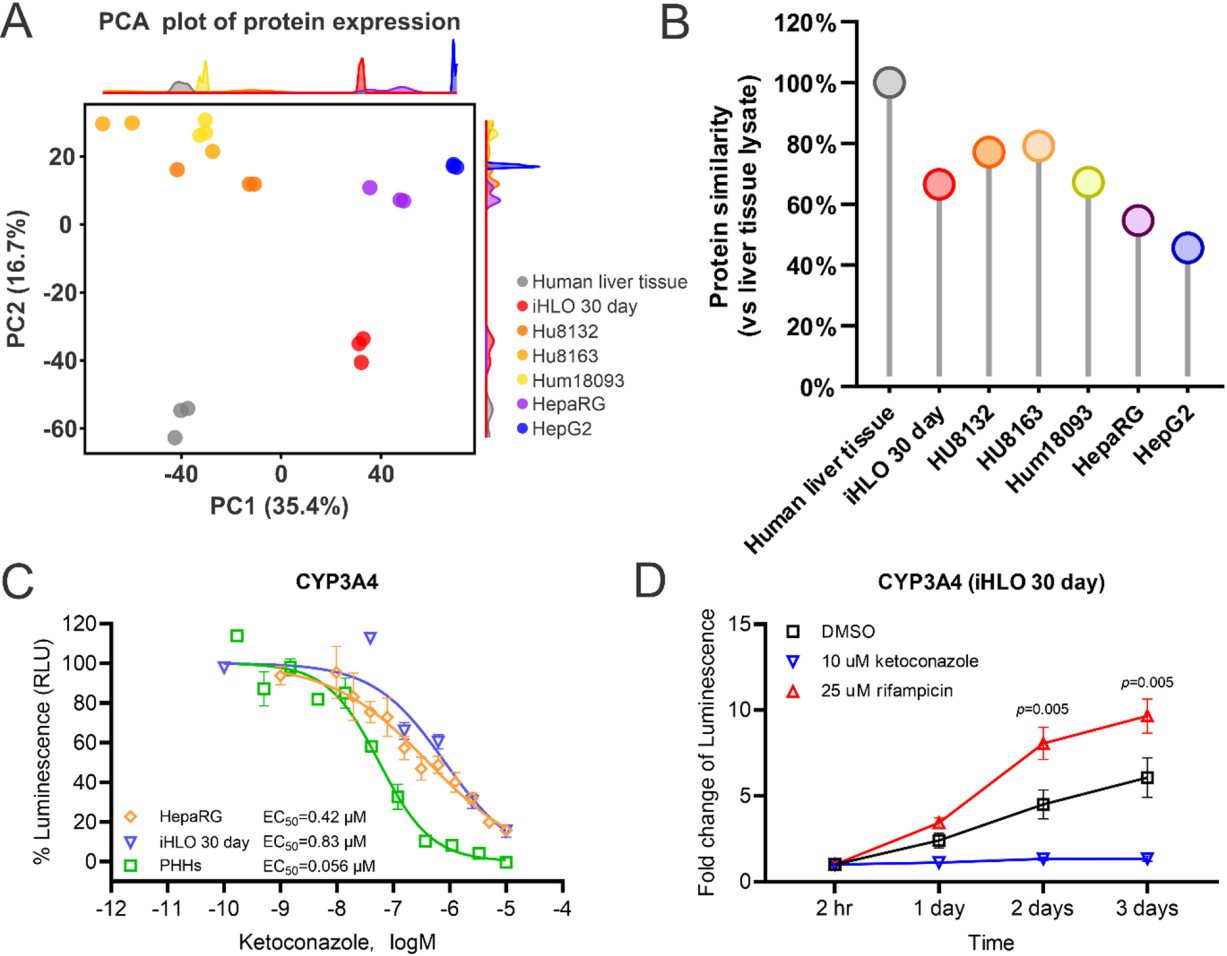
Suitability of iHLOs for toxicology applications. (**A**) Principal component analysis (PCA) with density plots of proteome data for each sample. (**B**) Protein expression pattern similarity comparison between Day 30 iHLOs and established liver models (PHHs, HepaRG cells, and HepG2 cells) relative to human liver tissue. (**C**) Concentration-response curves showing the inhibitory effect of ketoconazole on CYP3A4 activity in Day 30 iHLOs compared with HepaRG cells and PHHs. IC_50_ values are indicated for each model system. (**D**) Time-course analysis of CYP3A4 activity in Day 30 iHLOs over 72 h following treatment with the inducer rifampicin (25 µM) or the inhibitor ketoconazole (10 µM). All values represent mean ± standard deviation (*n* = 3 replicates). Statistical significance was determined using two-way analysis of variance (ANOVA) with Tukey’s multiple comparison test, and the p-value is shown in each graph.

To evaluate iHLOs as a model for toxicology studies, we compared proteomic profiles of Day 30 iHLOs, PHHs, HepG2 cells, and HepaRG cells against human liver tissue. Principal component analysis (PCA) indicated that Day 30 iHLOs exhibited a protein expression pattern more closely aligned with human liver tissue than HepaRG and HepG2 cells (Fig. 4A). Specifically, proteomics data showed that Day 30 iHLOs exhibited a protein expression similarity of 66.54% compared with human liver tissue, comparable to PHHs (67.26%–79.11%), but significantly higher than HepG2 (45.64%) and HepaRG cells (54.60%) (Fig. 4B).

Functional assays further validated iHLO for drug metabolism studies. CYP3A4 inhibition by ketoconazole yielded an IC_50_ of 0.83 µM in iHLOs, comparable to HepaRG cells (0.42 µM) but less potent than the IC_50_ (0.056 µM) from PHHs, demonstrating their applicability for studying CYP-mediated drug-drug interactions (Fig. 4C). Kinetic assays showed that rifampicin significantly induced CYP3A4 activity in iHLOs over three days, while ketoconazole consistently suppressed CYP3A4 activity over the same period (Fig. 4D). These results underscore the ability of iHLOs to respond predictably to enzyme inducers and inhibitors, positioning them as a robust alternative to PHHs for toxicology testing.

## Discussion

This study provides a comprehensive temporal proteomic analysis of iHLO maturation, identifying Day 30 as the optimal timepoint for application requiring drug metabolism and toxicology testing. By profiling the expression of 5,736 proteins across four developmental stages, the study delineates the dynamic progression of organoid maturation and establishes a temporal framework for selecting functional timepoints. Our differentiation protocol positions these four timepoints as critical for liver organoid development and maturation: Day 7 represents the hepatoblast differentiation stage, Day 15 marks the early hepatocyte stage, and Day 30 and Day 45 indicate distinct stages of liver organoid maturation towards achieving appropriate liver functions with an increasing population of liver cells. It is also noteworthy that Day 21, or around this timepoint, has been identified as an important stage for liver organoid functional development in several previous studies^13,15,28,29^. Future study will incorporate additional critical time points, including Day 21, into our analysis.

It should be acknowledged that current iHLO models are not yet ideal model, and significant differences exist between various differentiation protocols. Researchers should select the most suitable protocol based on their specific study objectives. This protocol was chosen due to its demonstrated capacity to generate iHLOs with high CYP enzyme activity, particularly CYP3A4, which is crucial for our subsequent compound or drug metabolism and toxicology evaluations. Despiste the wide variability in maturation timelines and functional benchmarks across different iHLO differentiation protocols^10,15,16,29–31^, our findings contribute a valuable reference for guiding hepatic organoid development, especially for optimizing their use in preclinical research and tailored applications such as drug metabolism and toxicology.

The findings from current study align with previous proteomic studies of liver organoids, which have demonstrated their capacity to recapitulate liver-specific phenotypes. For instance, Howell et al. quantified 4,405 proteins in murine hepatic organoids and observed differentiation-dependent upregulation of CYP450 enzymes, hepatic transporters, and other metabolic proteins, which parallels our observations in human iHLOs^14^. Similarly, Harrison et al. compare iHLO protein profiles with native liver tissue and highlighted enrichment of detoxification pathways in both systems, emphasizing the relevance of organoids for metabolically intensive applications^15^. This study adds to this body of work by providing a temporal dimension to proteomic profiling, demonstrating how maturity-dependent protein expression impacts organoid functionality. Importantly, the proteomic similarity of Day 30 iHLOs to PHHs, with a 33.46% expression difference from liver tissue, underscores their physiological relevance compared to traditional models like HepG2 cells, which exhibit a 54.36% protein difference from liver tissue, or HepaRG cells, with a 45.40% protein difference.

Unlike most prior studies, which typically analyze snapshots of organoid development at single timepoints^14,15^, this study employs a longitudinal approach to track the dynamic transitions of iHLO populations. The temporal profiling revealed critical insights into the developmental trajectory of hepatocyte and cholangiocyte differentiation, in addition to identifying increased cellular heterogeneity by Day 45. This trajectory mirrors aspects of human hepatic ontogeny, where hepatoblasts differentiate into functional hepatocytes and cholangiocytes before a subsequent phase involving mesenchymal cell expansion^32^. Interestingly, the mesenchymal shift by Day 45 suggests greater cellular complexity at later stages, potentially facilitating iHLO use in modeling liver pathologies such as fibrosis, cirrhosis, and liver remodeling, as indicated by increased expression of hepatic stellate cell markers such as DES and VIM^33,34^.

The peak functionality of iHLOs at Day 30, evidenced by maximal expression of CYP450 enzymes like CYP3A4, CYP2B6, and CYP2C9, revealed optimal conditions for xenobiotic metabolism studies. These enzymes are responsible for metabolizing 70% of clinically prescribed drugs^22,23^, establishing this timepoint as pivotal for investigating drug metabolism, interactions, and toxicity. The functional assays of CYP3A4 further validate this claim: iHLOs exhibited a concentration-dependent inhibition profile following ketoconazole treatment (IC_50_: 0.83 µM), comparable to HepaRG cells but distinct from PHHs. Although PHHs remain the gold standard for in vitro liver models, their availability, variability, and rapid dedifferentiation underscore the values of iHLOs as a scalable, reproducible alternative. In this context, we recognize the evolving landscape of in vitro liver models and intend to explore PHH spheroid models and co-culture spheroid models, which incorporate non-parenchymal cells, in future studies to further assess their functionality and to provide a comprehensive comparison with our iHLO model, thereby continuously advancing the field. The CYP3A4 induction and inhibition assays also highlight the potential of iHLOs for dynamic pharmacological testing, a key aspect of drug safety evaluations^35^.

Interestingly, the shift toward mesenchymal phenotypes at Day 45 highlights an unexplored research avenue. Hepatic stellate cells, characterized by DES and VIM expressions, are central to liver fibrosis and extracellular matrix remodeling^34,36^. The expansion of mesenchymal-like population at Day 45 suggests that these organoids could serve as an advanced model for studying fibrogenesis and testing anti-fibrotic therapies^34^. While most organoid research has focused on hepatocyte functionality and maturation, these findings illustrate the versatility of iHLOs across a range of applications, depending on the selected timepoint. Furthermore, the persistence of ALB expression despite declining hepatic protein levels supports the notion of functional heterogeneity, potentially reflecting a dynamic interplay between hepatocyte populations and expanding mesenchymal cells.

Despite its contributions, this study has limitations that warrant further exploration. First, the proteomic analyses did not detect markers for immune cells like Kupffer cells, which are critical for modeling immune-mediated liver diseases^37^. This common limitation^9^ underscores the need for future studies that incorporate immune components either *via* co-culture systems or through optimized differentiation techniques to promote immune cell development. Second, while CYP3A4 was extensively validated in functional assays, other key markers of liver functionality, such as bile acid transporters and detoxification enzymes, were not examined in as much detail. Addressing these gaps would provide a more holistic understanding of organoid functionality across different time points. Third, while bulk proteomics provides a comprehensive view of cellular differentiation pattern, more precise methods such as single-cell technologies (transcriptomics or proteomics)^38,39^ and spatiotemporal single-cell tracking^40,41^ would further elucidate the detailed cellular composition and 3D organization during iHLO development. Finally, although this study focused on a single induction protocol, variations in differentiation methods across research groups may affect maturation timelines, limiting the direct applicability of findings to alternate protocols.

The current study identified Day 30 of iHLOs as the optimal developmental stages that can be used for pharmacological and toxicological research. By providing a physiologically relevant alternative to 2D cultures and animal models, this study supports iHLO adoption for drug discovery and preclinical testing. Importantly, Day 45 organoids may offer added value for investigating liver pathologies involving mesenchymal interactions, such as fibrosis. The incorporation of patient-derived iPSCs into this system could further enhance its utility for personalized medicine, enabling the prediction of individual drug responses or disease progression^42^. By advancing the accuracy and scalability of in vitro liver models, this work contributes to reducing reliance on animal testing and promoting ethical innovation in preclinical research, as emphasized by Ingelman-Sundberg and Lauschke^43^.

In conclusion, the temporal proteomic profiling performed in this study defines a clear developmental roadmap for liver organoids, offering actionable insights for optimizing their use in drug metabolism, toxicological testing, and disease modeling. Further advancements, such as incorporating immune components or refining differentiation protocols, will likely bolster the versatility and translational relevance of this promising technology.

## Materials and methods

### Cell culture and compound treatment

HepG2 cells (ATCC, catalog: HB-8065) were cultured with 90% Eagle’s Minimum Essential Medium (EMEM, ATCC, catalog: 30-2003) and supplemented with 10% fetal bovine serum (FBS, Cytiva, catalog: SH30070.03) and 100 U/mL penicillin-100 µg/mL streptomycin (Thermo Fisher Scientific, catalog: 15140122).

Fully differentiated HepaRG cells (Lonza, catalog: NSHPRG) were plated with 90% of William’s E Medium (Thermo Fisher Scientific, catalog: A1217601) and supplemented with 10% HepaRG thawing and plating medium supplement (Lonza, catalog: MHTAP) and 25 U/mL penicillin-25 µg/mL streptomycin. After overnight incubation, the medium was changed to 90% of William’s E Medium and supplemented with 12.5% HepaRG preinduction and toxicity medium supplement (Lonza, catalog: MHPIT) and 25 U/mL penicillin-25 µg/mL streptomycin, followed by 72 h incubation. For compound treatment, the medium was replaced by 90% of William’s E Medium supplemented with 12.5% HepaRG preinduction and toxicity medium supplement, 0.6% HepaRG induction medium supplement (Lonza, catalog: MHIND), and 25 U/mL penicillin-25 µg/mL streptomycin.

Plateable PHHs Hu8132 and Hu8163 (Thermo Fisher Scientific, catalog: HMCPMS) were thawed in cryopreserved hepatocyte recovery medium (Thermo Fisher Scientific, catalog: CM7000) and plated with William’s E Medium supplemented with primary hepatocyte thawing and plating supplements (Thermo Fisher Scientific, catalog: CM3000). After overnight incubation, the medium was replaced with William’s E Medium supplemented with primary hepatocyte maintenance supplements (Thermo Fisher Scientific, catalog: CM4000) and incubated for 24 h before compound treatment. Additional plateable PHHs Hum180931 (Lonza, catalog: HUCPI) were thawed and plated in hepatocyte plating medium (Lonza, catalog: MP250) overnight, then the medium was replaced with hepatocyte culture medium (Lonza, catalog: CC-3198).

### iPSC-derived human liver organoid culture

The iHLOs at various developmental stages were obtained from 3Dnamics, Inc. (now part of Dexorgen Inc., Rockville, MD, USA), with their proprietary protocol adapted from published literature^18–21^. Briefly, human iPSCs were reprogramed from wild-type human foreskin fibroblasts (ATCC, catalog: CRL-2522)^44,45^ using a non-integrating Sendai virus (CytoTune™-iPS 2.0 Sendai Reprogramming Kit, Thermo Fisher Scientific, catalog: A16517) and maintained in StemFlex™ Medium (Thermo Fisher Scientific, catalog: A3349401) with medium changes every other day^46^. For iHLO differentiation, iPSCs were dissociated with Versene (Thermo Fisher Scientific, catalog: 15040066) on Day 0 and plated into an AggreWell plate (STEMCELL Technologies, catalog: #34421) in Stemflex Medium (Thermo Fisher Scientific) to form aggregates, which were then transferred to an ultralow attachment dish on Day 1 and cultured in RPMI1640 supplemented with B27 supplement minus insulin (RPMI/B27-Insulin), CHIR99021 (1 µM), Activin A (100 ng/mL), WNT3a (50 ng/mL), and Rock Inhibitor (Y27632, 5 µM). On Day 2, the medium was switched to RPMI/B27-Insulin with Activin (100 ng/mL) and Hepatocyte Growth Factor (HGF, 20 ng/mL). From Days 3 to 8, the medium was switched to Knockout DMEM, 20% Knockout Serum Replacer, beta-mercaptoethanol (BME, 100 µM), 1% Glutamax, non-essential amino acids, and 1% DMSO. From Days 9 to 16, the medium was switched to hepatozyme (Thermo Fisher Scientific) supplemented with Glutamax, HGF (20 ng/mL), and Ocostatin M (20 ng/mL). From Day 17 onward, liver organoids were matured in Leibovitz L15 medium supplemented with Dihexa (100 nM), Dexamethasone (100 nM), Hydrocortisone (10 µM), Sodium L-ascorbate (50 µg/mL), Insulin-transferrin-selenium (0.58%), FBS (8.3%), Glutamax, and BME. Liver organoids were maintained in a humidified 37 °C, 5% CO_2_ incubator.

### Preparation of protein lysates

The cells collected from 6-well plates were lysed in 0.4 ml lysis buffer (20 mM Tris-HCl, pH 7.5, 150 mM NaCl, and 2% SDS) followed by sonication (10 cycles of 10 s pulses with 10 s rest intervals) using a Fisher Scientific Sonic Dismembrator Model 500 at 15% amplitude. Lysates were centrifuged at 15,000 × g for 10 min at 4 °C. Supernatants were collected and stored at -80 °C for further analysis. Protein concentration was determined using a BCA Reducing Reagent compatible assay kit (catalog: 23250, Thermo Fisher Scientific).

### Tandem mass tag (TMT) labeling

The TMT18-plex labeling method was used to tag samples with different mass isotopes in one batch. Briefly, 100 µg of protein lysate from each sample was subjected to in-solution trypsin digestion. Pre-digestion samples along with digested samples were run on SDS-PAGE and stained to verify digestion efficiency. Isobaric labeling was performed using a TMT-18plex kit. Labeled peptides were combined, dried in a vacuum concentrator, and stored at -80 °C.

### Fractionation of labeled peptides by basic reversed-phase ultra-high performance liquid chromatography (UHPLC)

Dried labeled peptides were resuspended in 10 mM triethylammonium bicarbonate (TEABC) buffer. Labeling efficiency was determined by analyzing a small aliquot (1%) of the sample, with a minimum 95% efficiency required for further processing. Fractionation of TMT-18plex-labeled peptide mixture was performed using an Agilent AdvanceBio Column (2.7 μm, 2.1 × 250 mm) with an Agilent UHPLC 1290 system. Separation was achieved using a gradient of Solvent B (10 mM TEABC, pH 8.0, 90% ACN) and Solvent A (10 mM TEABC, pH 8.0) at the flow rate of 250 µL/min. Eluted fractions were collected into a 96-well plate using a 1260 series auto-sample fraction collector and further combined into 24 fractions for liquid chromatography–tandem mass spectrometry (LC-MS/MS) analysis.

### Nanospray LC-MS/MS analysis and database search

LC/MS/MS analysis was performed using a Thermo Fisher Scientific Orbitrap Exploris 240 Mass Spectrometer and a Thermo Dionex UltiMate 3000 RSLCnano System. Each peptide fraction was loaded onto a peptide trap cartridge at 5 µL/min. Trapped peptides were eluted onto a reversed-phase 25 cm C18 EasySpray nano column (Thermo Fisher Scientific) using a linear gradient of acetonitrile (3–36%) in 0.1% formic acid over 110 min at 0.3 µL/min. Eluted peptides were ionized and sprayed into the mass spectrometer using an EasySpray Ion Source (Thermo Fisher Scientific) with a spray voltage of 1.6 kV and a capillary temperature of 275 °C.

The Exploris 240 instrument was operated in a data-dependent mode, alternating between full scan MS and MS/MS acquisition. Survey full scan MS spectra (m/z 350 − 1800) were acquired in the Orbitrap with 60,000 resolutions (m/z 200) after accumulating ions to a 3 × 10^6^ target value based on predictive automatic gain control (AGC). The maximum injection time was set to 100 ms. The 20 most intense multiply charged ions (z ≥ 2) were sequentially isolated and fragmented in the octopole collision cell by higher-energy collisional dissociation (HCD) using normalized collision energy of 32 with an AGC target of 1 × 10^5^ and 200 normalized AGC targets (%) at 45,000 resolutions. The isolation window was set to 0.7, and the fixed first mass was 120 m/z. Dynamic exclusion was set to 20 s, with charge state screening enabled to reject unassigned and 1+, 7+, 8+, and > 8+ ions.

### MS peptide assignment

Five sets of 24 MS raw data files acquired from analysis of 24 fractions from each set are searched against the human protein sequences database obtained from the UniprotKB website using the Proteome Discoverer 2.4 software (Thermo, San Jose, CA) based on the SEQUEST and percolator algorithms. The false positive discovery rates (FDR) were set at 1%. The resulting Proteome Discoverer Report contains all assembled proteins with peptide sequences and peptide spectrum match counts (PSM#) and TMT-tag-based quantification ratios. Relative protein abundance was calculated as the ratio of abundance determined by the TMT tags. A normalization of the ratio (relative abundance) was performed by using the summed reporter ion intensities.

### Proteomic data analysis

The mass spectrometry proteomics data from this study have been deposited to the ProteomeXchange Consortium *via* the PRIDE partner repository with the dataset identifier PXD063438. Protein expression trends across four developmental time points were analyzed using Short Time-series Expression Miner (STEM, version 1.3.13)^47^ with normalized protein counts. Six groups of proteins were classified based on expression pattern. Differentially expressed proteins were selected using default settings. Gene Ontology (GO) and Kyoto Encyclopedia of Genes and Genomes (KEGG) pathway enrichment analysis were performed using The Database for Annotation, Visualization, and Integrated Discovery (DAVID)^48^. Significant enriched biological pathways were selected using p-value < 0.05. Proteins with significant differential expressions between groups were identified by Student’s t-test (|log ratio| > 0.585 and p-value < 0.05). The percentage of changed proteins was calculated by comparing each group to the human liver tissue sample. Protein expression pattern similarity was quantified as 100% - (% of significantly differentiated proteins). Principal component analysis (PCA) and clustering heatmaps were performed using R (version 4.5.0).

### Histological analysis

iHLOs were preserved in a 30% sucrose solution and then embedded in optimal cutting temperature (OCT) compound for cryosection. Hematoxylin and eosin (H&E) staining (VitroVivo Biotech) was performed to evaluate the structure of iHLOs on Day 30. Briefly, slides were immersed in 4% paraformaldehyde (Thermo Fisher Scientific, catalog: J19943.K2) immediately after removal from the freezer for 1 min, then washed with water. Nuclei were stained by immersing the slides in a Gill 2 hematoxylin solution (MilliporeSigma, catalog: GHS232-1 L) for 3 min, followed by washing in water and immersing in a bluing agent (0.1% NaHCO_3_) for 1 min, then washing in water. Slides were briefly immersed in 95% ethanol, then cytoplasm was stained by immersing in Eosin (MilliporeSigma, catalog: 318906-500ML) for 1 min and quickly dipping once in water. Samples were dehydrated by immersing in two changes of 95% ethanol and then in three changes of 100% ethanol for 10 dips each. Slides were immersed in two changes of xylene for 10 dips each and mounted with Permount slide mounting medium (Thermo Fisher Scientific, catalog: SP15-100) using glass coverslips.

### Whole mount immunostaining

iHLOs in culture medium were transferred into a 1.5 ml tube and washed twice with PBS. Organoids were fixed in 4% paraformaldehyde (Electron Microscopy Sciences, catalog: 15714-S) for 30–45 min at room temperature with shaking. After three PBS washes, iHLOs were incubated in 100 µL Ce3D permeabilization/blocking buffer (BioLegend, catalog: 427706) for 1 h at room temperature. iHLOs were then incubated with the primary antibody solution diluted in antibody dilution buffer (Biolegend, catalog: 427708) overnight at 4 °C with shaking. After removing primary antibody and washing three times with PBS, iHLOs were incubated with secondary antibody diluted in antibody dilution buffer for 2 h at room temperature. Following three PBS washes, iHLOs were transferred to 96-well plates for imaging using the Opera Phenix Plus High-Content Screening System (Revvity). The following antibodies were used: cytokeratin 7 (CK7/KRT7) (Abcam, ab181598, 1:1000), CYP3A4 (Invitrogen Antibodies, MA5-17064, 1:1000), desmin (R&D Systems, AF3844, 1:20), and albumin (Fortis Life Sciences, A80-129 A, 1:1000). The secondary antibodies are highly cross-adsorbed Alexa Fluor™ Plus conjugates: Donkey anti-Rabbit IgG (H + L) 488 (Thermo Fisher Scientific, catalog: A32790), Donkey anti-Goat IgG (H + L) 647 (Thermo Fisher Scientific, catalog: A32849), and Donkey anti-Mouse IgG (H + L) 594 (Thermo Fisher Scientific, catalog: A32744).

### P450-Glo™ CYP3A4 activity assay

CYP3A4 activity was measured using the P450-Glo™ CYP3A4 assay kit (Promega, catalog: V9001). HepaRG cells (8000 cells/25 µL/well) or PHHs (Hu8163, 17,500 cells/25 µL/well) were seeded in collagen I-coated 384-well white flat-bottom plates (Corning, catalog: 354665). Three 30-day iHLOs of similar sizes (40 µL/well) were transferred to a 384-well ultra-low attachment plate (Corning, catalog: 4516) and incubated for 24 h before testing.

For CYP3A4 inhibition testing, ketoconazole (MilliporeSigma, catalog: PHR1385) was prepared at 2× final concentration in culture medium and added in equal volume to each well of a 384-well plate for 24 h. After treatment, the medium was removed, and 20 µL/well of fresh medium containing luminogenic CYP substrate was added, followed by a 1-hour incubation at 37 °C. Then, 20 µL of Luciferin Detection Reagent was added per well and incubated for 30 min. Luminescence was measured using a ViewLux plate reader (Perkin Elmer), with data reported as relative luminescence units.

For kinetic CYP3A4 activation and inhibition testing, ketoconazole or rifampicin (MilliporeSigma, catalog: R7382) was prepared at 2× final concentration in culture medium containing 2× luminogenic CYP substrate. Equal volumes were added to each well and incubated for 24, 48, and 72 h separately. At each time point, 20 µL of iHLO culture medium was transferred to a 384-well white flat-bottom plate, 20 µL of Luciferin Detection Reagent was added, and the mixture was incubated for 30 min before measuring luminescence.

### Statistical analysis

All data were presented as mean ± standard deviation (SD) from at least three independent experiments unless otherwise stated, and concentration response curves were plotted using GraphPad Prism software. The two-tailed unpaired t-tests were used for single comparisons, and the analysis of variance (ANOVA) with Tukey’s multiple comparison test was used for multiple comparisons. Differences were considered statistically significant at p-value < 0.05.

## Supplementary Information

Below is the link to the electronic supplementary material.

Supplementary Material 1

## Data Availability

Original data generated and analyzed during this study are included in this published article, the supplementary material, or are available from the corresponding authors on reasonable request. The mass spectrometry proteomics data from this study have been deposited to the ProteomeXchange Consortium *via* the PRIDE partner repository with the dataset identifier PXD063438. (link: (https://www.ebi.ac.uk/pride); Project accession: PXD063438; Token: Zazd43aaeRyC)
