# Supplementary material for: ​Temporal proteomic profiling of iPSC-derived human liver organoids reveals optimal maturation for drug metabolism and toxicology
Source: Sci Rep. 2026 Jan 14;16:2757. doi: 10.1038/s41598-025-32539-0 (PMC12824400; doi:10.1038/s41598-025-32539-0)
Supplement: Supplementary file 1 — Supplementary Material 1 [file 41598_2025_32539_MOESM1_ESM.zip › Supplementary Figures.pdf]

Supplementary Figure 1

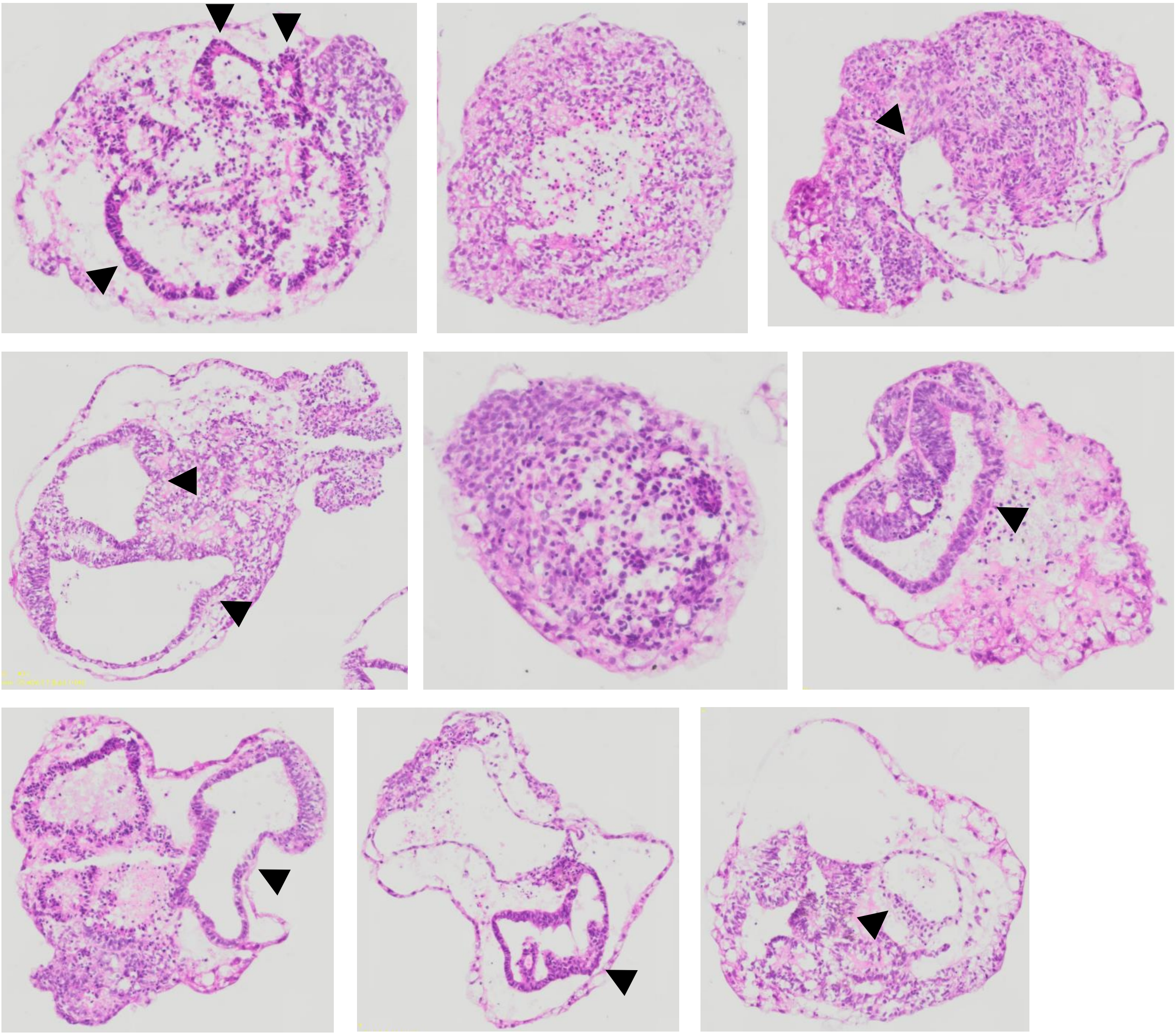

Supplementary Figure 1. Histological Structure and Luminal Formations in Day 30 iHLOs. Additional H&E staining images of Day 30 iHLOs, showing the histological structure formation. Arrowheads indicated luminal structure in the iHLOs.

Supplementary Figure 2

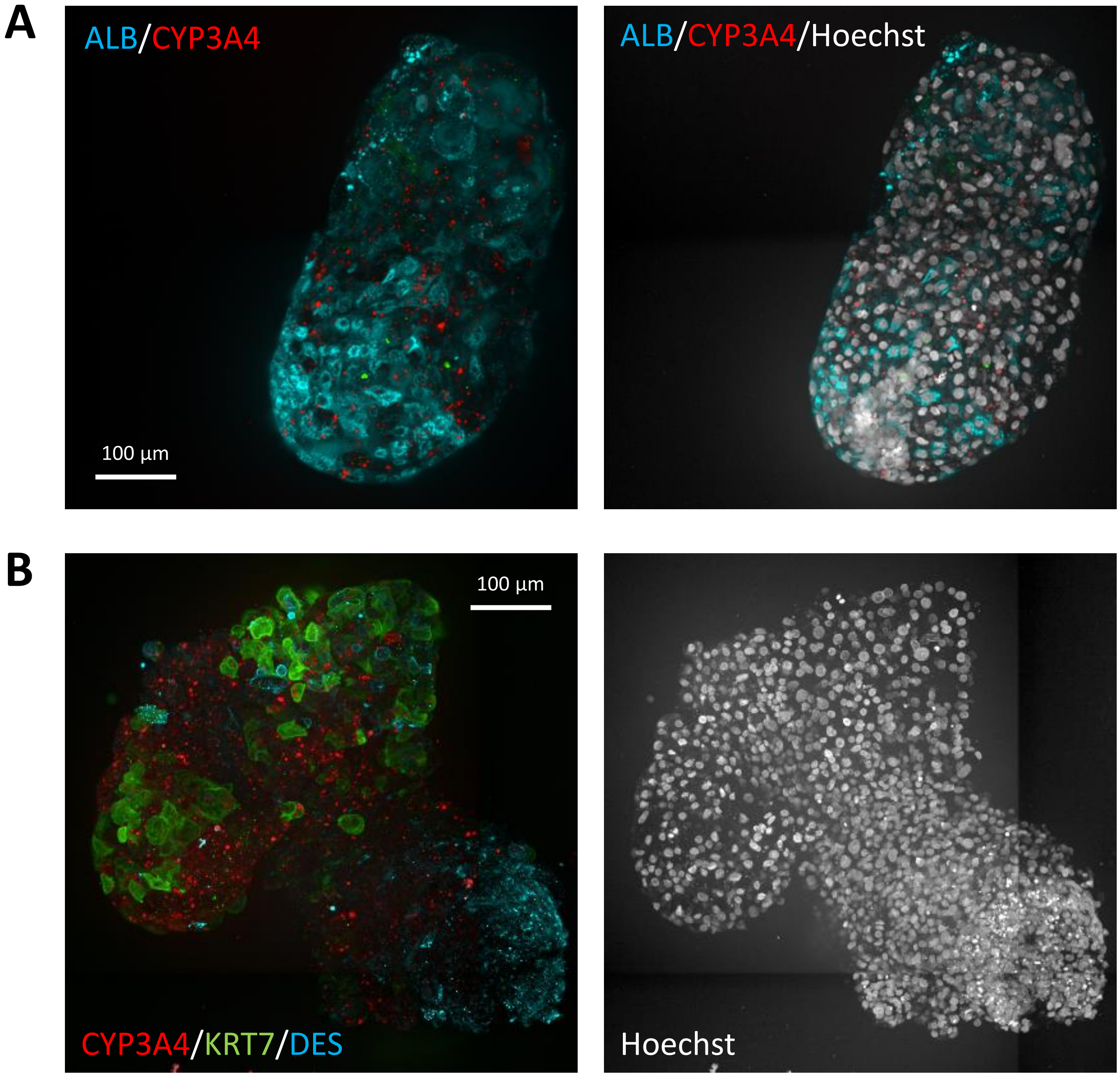

Supplementary Figure 2. Multi-lineage Co-differentiation in iHLOs Visualized by Immunostaining. (A) Whole mount immunofluorescence staining of Day 30 iHLOs showing ALB (cyan) and CYP3A4 (red) localization. (B) Whole mount immunofluorescence staining of Day 45 iHLOs showing CYP3A4 (red), KRT7 (green), and DES (cyan) demonstrating co-differentiation of multiple cell lineages.

Supplementary Figure 3

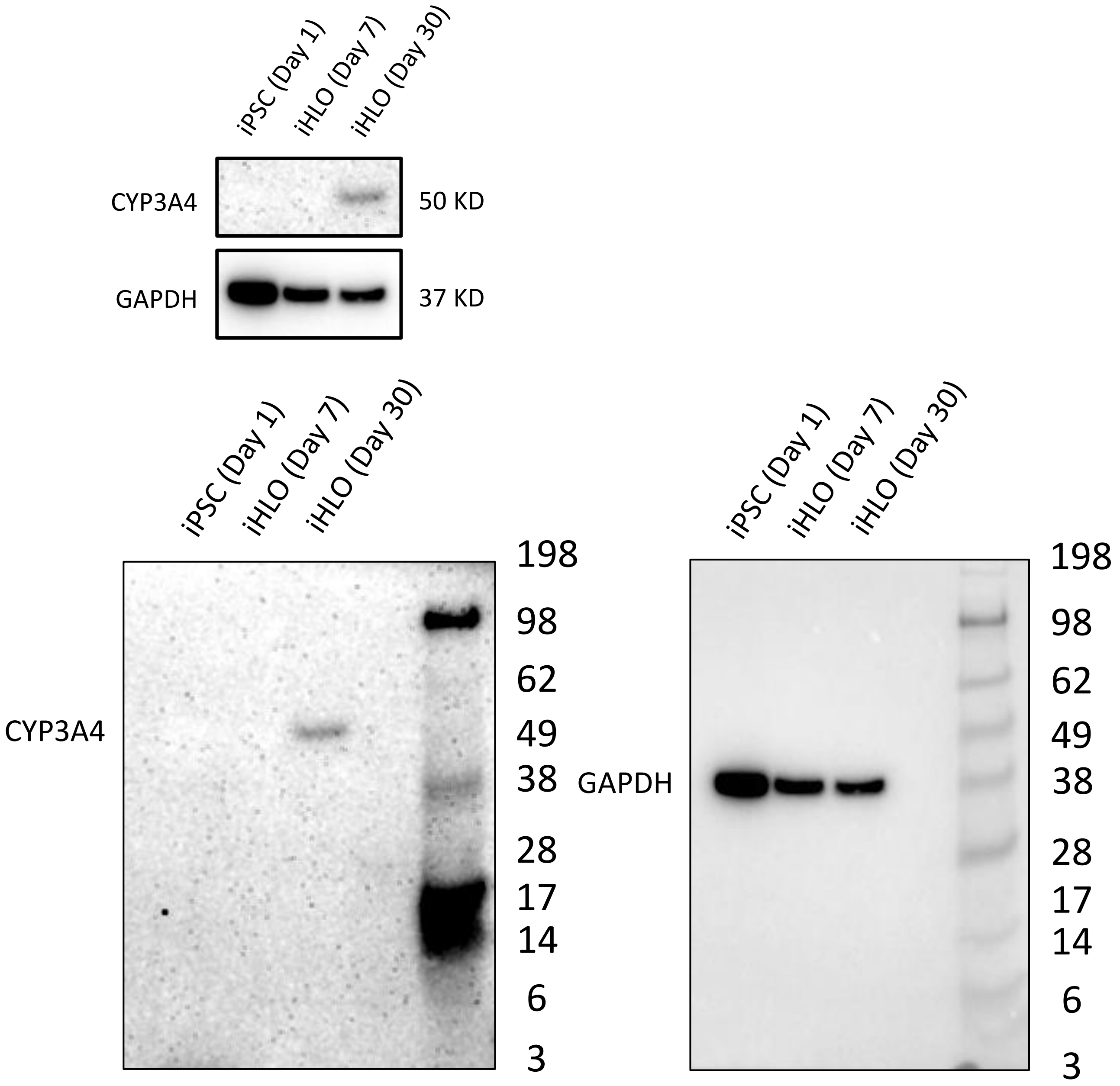

Supplementary Figure 3. Temporal expression of CYP3A4 in iHLOs by Western blot. Western blot shows the expression of CYP3A4 in iHLOs on Day 1, Day 7, and Day 30.

Method for Western blotting

iHLO samples were lysed in RIPA buffer (Cell Signaling Technology) supplemented with protease inhibitors (cOmplete ULTRA Tablets, EDTA-free, Roche) and phosphatase inhibitor cocktail (PhosSTOP, Roche). The lysates were centrifuged at 16,000 rpm for 30 min. Supernatant was collected for protein quantitation with a BCA assay kit (Pierce BCA Protein Assay Kit, ThermoFisher Scientific). The supernatant with similar protein concentrations were subsequently applied to Bis-Tris gels for protein separation. Proteins were transferred to polyvinylidene difluoride (PVDF) membrane by dry transfer (iBlot 2 Gel Transfer Device, both from ThermoFisher Scientific). Immunoblot analysis was performed with indicated antibodies (CYP3A4: #13384, Cell Signaling Technology; GAPDH: #5174, Cell Signaling Technology) and the chemiluminescence signal was visualized with Luminata Forte Western HRP substrate (EMD Millipore) in a BioSpectrum system (UVP, LLC).

Supplementary Figure 4

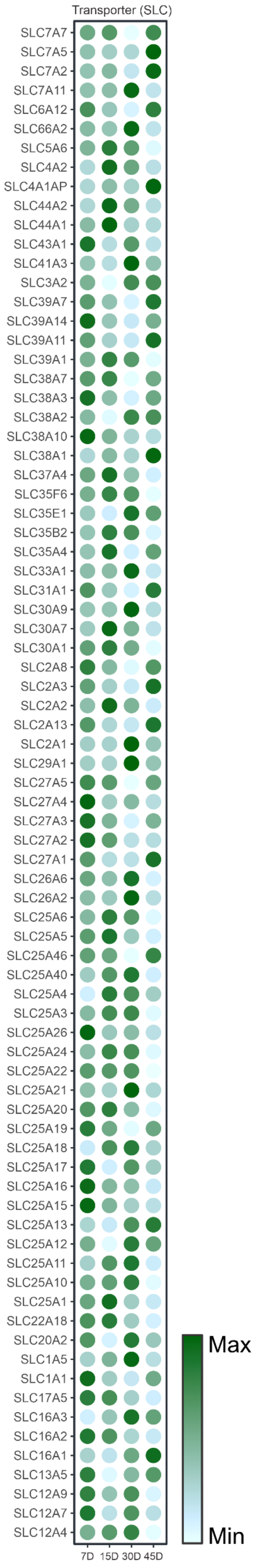

Supplementary Figure 4. Developmental dynamics of 79 SLC family transporters in iHLOs. Bubble plot depicting the expression profiles of 79 solute carrier (SLC) family transporters across four developmental stages of iHLOs (Days 7, 15, 30, and 45). Bubble color intensity indicates relative protein abundance.
